# Communication experiences of family caregivers of hospitalized adults with intellectual and developmental disabilities—A qualitative study

**DOI:** 10.1002/nop2.557

**Published:** 2020-07-06

**Authors:** Marie Lourdes Charles

**Affiliations:** ^1^ College of Health Professions Lienhard School of Nursing Pace University New York New York USA

**Keywords:** adults, developmental disability, family caregivers, hospitalization, intellectual disability, nurses, nursing

## Abstract

**Aim:**

To explore communication experiences between family caregivers of adults with intellectual and developmental disabilities (I/DD) and healthcare personnel during hospitalization.

**Design:**

A qualitative descriptive study approach with interviews of family caregivers was used.

**Method:**

Face‐to‐face, semi‐structured interviews were conducted from June–September 2015 with ten family caregivers of adults with I/DD. Participants were recruited through an advocacy organization in the north‐eastern United States. Data were analysed by content analysis. The Standards for Reporting Qualitative Research was the chosen checklist.

**Results:**

The four overarching themes: “Need for Advocacy”; “Need for Better Communication”; “Sense of Abandonment”; and “Lack of Confidence” along with 12 subthemes were identified. Overall, participants reported miscommunications leading to instances of mistrust in hospital staff's competence to deliver quality patient care.

## 1 INTRODUCTION

Americans with intellectual and developmental disabilities (I/DD) are living longer due to healthier lifestyles, health promotion, diagnostic examinations and medical interventions (Vincent & Velkoff, 2010). Longer lifespans translate into higher risks for hospitalization for adults with I/DD. Multiple comorbidities and communication impairments result in poor patient outcomes and increase the risk for rehospitalization (Hemsley et al., 2016). Loss of control, fear of negative outcomes, restrictive medical equipment, increased environmental stimuli and longer than average hospital stays are some of the obstacles confronted by individuals with I/DD (Bradbury‐Jones, Rattray, Jones, & McGillivray, 2013; Wark, Hussain, & Edwards, 2014; Welch & Barksby, 2011). Therefore, adults with I/DD often rely on family caregivers to be advocates and communicators.

Individuals with I/DD have different levels of communication challenges; nevertheless, they do communicate. Communication patterns employed by these individuals may be categorized as pre‐symbolic, symbolic or verbal (Boardman, Bernal, & Hollins, 2014). At least 80% of individuals with I/DD have communication impairments; some use pre‐symbolic methods and depend on others to anticipate their needs; 60% use symbolic methods such as pictures and signs to communicate (Emerson, 2001). Caregivers serve as surrogate communicators because of their in‐depth knowledge of their loved ones, including communication patterns. Persons with I/DD often rely on family caregivers to provide hospital personnel with their health history, care needs and guidance about level of functioning (Hemsley & Balandin, 2014). Consequently, caregivers' interactions with hospital personnel may have a great on patient safety and satisfaction.

Caregiver participation in the care of their loved ones is relevant to all populations, especially for adults with I/DD. This is especially true for adults with I/DD, as they rely on caregivers more than the average population because of communication challenges. There are negative implications for patient care outcomes if caregivers and hospital personnel do not communicate effectively. Generally, research about the experiences of caregivers of adult patients with I/DD is limited (Lunsky, Tint, Robinson, Gordeyko, & Ouellette‐Kuntz, 2014).

Exploring the communication experiences of family caregivers of adults with I/DD during hospitalization, through this qualitative descriptive study, allowed caregivers to voice their perspectives on interactions with hospital staff. The present study contributes to the literature in that it is from the perspective of caregivers who had a hospitalized adult family member with I/DD. In addition, the results of this study have the potential to have an impact on nursing practice.

## 2 BACKGROUND

During hospitalization, problems communicating with patients and families have an impact on the following: (a) obtaining medical histories; (b) treatment plans; (c) patient satisfaction; (d) diagnostic procedures; (e) health prevention measures; (f) accessibility; and (g) attitudinal barriers (Minihan et al., 2011). Interactions between hospital staff and adults with I/DD were a common concern in the literature. However, there is a paucity of research on caregivers of adults with I/DD during acute care hospitalization.

In a study of 35 individuals with cognitive and physical disabilities, who could advocate for themselves, participants described nursing care received during hospitalization (Smeltzer, Avery, & Haynor, 2012). They reported poor communication with nursing staff, lack of competence and negative attitudes as reasons for receiving inadequate care. Ineffective care included not being provided with the extra time persons with disabilities require to eat, bathe and get out of bed nor were they given adequate time for questions and answers. The study also found assessment deficiencies such as hospital staff not being able to identify the degree of patients' communication deficits. Based on the study's outcome, persons with disabilities may be at higher risk for injury whether they can advocate for themselves or not.

Studies have revealed that communication issues between these adults and hospital personnel may contribute to negative staff attitudes, poor health outcomes and increased risk for complications (Smeltzer et al., 2012). Ailey, Johnson, Fogg, and Friese (2015) indicated that adults with intellectual disabilities were twice as likely to have complications following a surgical procedure and were nearly four times as likely to have complications if they had been diagnosed with cerebral palsy, autism spectrum or aggressive behaviour.

Several other studies accentuated a lack of original research on adults with I/DD in the acute care setting. Bigby (2008) conducted a study about the health of adults with I/DD; however, the participants were living in the community instead of during a hospitalization. Although Bradbury‐Jones et al. (2013) focused on the acute care setting, their approach was a literature review. Furthermore, much of the research, similar to Hemm, Dagnan, and Meyer (2015), had been conducted in the United Kingdom and Australia limiting the applicability of the results because of different healthcare systems and geographic locations.

Lewis and Stenfert‐Kroese (2010) concentrated on the experiences of nurses caring for individuals with intellectual disability. Miller et al. (2016) investigated the hospital experience of patients and their informal caregivers. Their findings about caregivers were applicable to the general patient population and not specific to adult patients with I/DD. The literature indicated the need for further investigation on adults with I/DD and caregivers (Bigby, 2008; Bradbury‐Jones et al., 2013; Hemm et al., 2015; Lewis & Stenfert‐Kroese, 2010; Miller et al., 2016).

Patients and family caregivers are often viewed as a unit because of their physical, emotional, psychological, financial and social history (Stavrou et al., 2017). Omitting caregivers from the treatment plan prevents them from sharing valuable health information (Wolff, Spillman, Freedman, & Kasper, 2016). Facilitating caregivers to act as surrogate communicators can minimize negative outcomes. Hence, this study examined how family caregivers of hospitalized adults with I/DD perceived their communication experiences with healthcare personnel.

## 3 THE STUDY

### 3.1 Design

The research question guiding this study was: How did family caregivers of hospitalized patients with intellectual and developmental disabilities perceive their communication experiences with healthcare personnel? The study provided family caregivers a voice in describing their interactions with hospital staff. A qualitative descriptive approach was used to gather responses to open‐ended questions and probes from the family caregivers' perspective (Colorafi & Evans, 2016).

### 3.2 Method

Recruitment occurred through an advocacy organization for individuals with I/DD. An invitation to participate was posted on the organization's website. Potential participants contacted the investigator by telephone or email. Inclusion criteria were as follows: (a) persons who had been family caregivers of an adult with I/DD for at least 3 years; (b) the individual with I/DD was 30 years or older at the time of hospitalization; (c) hospitalization was overnight or longer in the past 3 years; (d) caregivers were fluent in English; and (e) caregivers resided in a large metropolitan area in the north‐eastern United States. Participants shared the common experience of being family caregivers of adults with I/DD who had been hospitalized; therefore, homogenous sampling was applicable (Etikan, Musa, & Alkassim, 2016). Unpaid caregivers of diverse backgrounds, ethnicities and educational levels, 30 years of age or older were selected as participants. Age 30 was a criterion for both participant and patient because the Centers for Medicare and Medicaid Services consider anyone up to the age of 26 eligible for dependent coverage (Centers for Medicare & Medicaid Services, 2014). As such, anyone age 30 is an adult. A timeframe of 3 years was chosen based on the investigator's experience caring for families in the community.

Fifteen caregivers volunteered to participate in the study. Two volunteers were not selected because the relatives with I/DD were below the age of 30. Three did not qualify because the relative's hospitalization took place more than 3 years prior to the study. A total of 10 caregivers were selected consisting of five parents, three siblings and two siblings‐in‐law. Six females and four males participated. They were 60% Caucasian, 30% Black/African American and 10% Hispanic. The demographic information for participants is shown in Table 1. Caregivers were 60–82 years old with a mean of 72.1. At the time of hospitalization, the patients were between 35–62 years old with a mean of 50.4, consisting of two females and five males. The time between patient hospitalization and caregivers' interviews is included in Table 1 and ranged from 6 months–3 years with a mean of 1.71.

**Table 1:** Study participants demographics (*N* = 10)

		*M*	*SD* (Range)
Age		72.1	7.4 (60–82)
Time (in years) between patient hospitalization and caregivers' interviews	Parents	1	0.7 (0.5–3)
	Sibling	2.3	
	Sibling‐in‐law	2.5	

		*N*	%
Gender	Female	6	60
	Male	4	40
Race	Caucasian	6	60
	Hispanic	1	10
	Black	3	30
Relationship status	Parent	5	50
	Sibling	3	30
	Sibling‐in‐law	2	20

Abbreviations: *M*, mean; *N*, sample size; *SD*, standard deviation.

Interviews were conducted between June–September 2015 and took place in private homes or offices based on the caregiver's choice. Each caregiver received a consent form via postal service or email 2 weeks prior to scheduling the interview. Each interview began with the investigator reviewing the consent. Risks and benefits of taking part in the study were reviewed with caregivers. The investigator answered the caregiver's questions and witnessed the caregiver signing the consent form. In addition, the caregiver filled out a demographic questionnaire which is available in Appendix S1. Confidentiality was established by using a set of numbers chosen by caregivers to identify themselves. Initials unrelated to caregivers' names were used during the interviews. All data were kept in a locked cabinet in the investigator's office.

Using a face‐to‐face and semi‐structured format allowed the investigator to observe cues from interviewees to explore topics further. In‐depth questions and probes facilitated caregivers' revelations of their lived experiences and perceptions about their communication experiences with healthcare personnel. Lastly, field notes on body language, facial expressions and non‐verbal cues were recorded in the investigator's journal.

Each interview was audiotaped and lasted approximately 45 min. After eight interviews, saturation was reached. Two additional interviews were conducted to ensure all relevant factors were included. A professional transcriptionist transcribed the audiotaped interviews. Individual findings were mailed to each caregiver for review prior to a follow‐up telephone call by the investigator. Telephone calls lasted 10–15 min. This process of member checking presented caregivers the opportunity to elaborate or clarify interpretations. Caregivers agreed that reports reflected their views and requested no changes.

Components of the study such as topic, context, data collection process, analysis and selection of participants were structured to meet the criteria for credibility, dependability and confirmability (Guba & Lincoln, 1989). To assure credibility, an outside disinterested researcher who was an expert in qualitative methods evaluated the study. Dependability was addressed through the investigator maintaining detailed notes and rich descriptions of the study format, data collection and analysis. A reflexive journal ensured the study's confirmability. Caregivers who shared the common experience of being family caregivers for adults with I/DD who had been hospitalized were selected, thereby increasing the probability of discovering elements most relevant to the issue (Guba & Lincoln, 1989). Detailed descriptions of methodology, procedures and data collection satisfied the dependability criteria, thus allowing the study to be replicated in the future (Guba & Lincoln, 1989). Standards for Reporting Qualitative Research (O'Brien, Harris, Beckman, Reed, & Cook, 2014) were used to ensure transparency as shown in Supplementary File 1.

### 3.3 Ethics

Institutional Review Board (IRB) approval was obtained in 2015 from Nova Southeastern University (NSU No. 05061504). The investigator followed the ethical guidelines of the Collaborative Institutional Training Initiative. This is a programme which educates researchers on ethics and regulatory oversight governing responsible conduct in research.

### 3.4 Analysis

Conventional content analysis was employed. According to Hsieh and Shannon (2005), conventional content analysis is appropriate to describe a phenomenon when existing theory or research on the phenomenon is limited. The investigator read the text several times to obtain a general sense of the data, to gain new insight and extracted categories from the data.

Transcripts were read several times before categorizing the data. The investigator extracted meaning from statements, words, phrases and sentences. Recurrent words or phrases were grouped together. The unit of analysis was phrases representing the smallest segment of text reflecting a participant's meaning (Elo & Kyngas, 2008). Phrases were initially labelled as codes (Creswell, 2002/2012). Phrases with similar meaning were grouped according to descriptions to form in vivo codes. A codebook of 16 subthemes was developed (Fonteyn, Vettese, Lancaster, & Bauer‐Wu, 2008). Potential themes were identified during this phase. The final list was condensed, and subthemes reduced to fewer themes. A mentor also reviewed the data to confirm that categories, themes and subthemes were consistent.

## 4 RESULTS

Quotes from caregivers were analysed. Four themes and 16 subthemes were generated and formatted into Table 2.

**Table 2:** Themes and subthemes

Themes	Subthemes	Quotes (Participant)
Need for Advocacy	Have to be there	“He is not able to tell nurses or doctors anything. So, when he is hospitalized…someone has to be with him, so nine times out of ten it's me.” (Mrs. A)
		“Well it's fear, there's a sense of fear that perhaps something bad can happen, you know, maybe Anna's not getting the proper care, either through diagnosis or whatever and there's this … it could have a bad outcome.” (Mr. F)
		“Really if she hadn't been in a residence and I had to place her in the hospital I would've had to stay there.” (Mrs. B)
	They don't ask	“They never asked, they never asked about it; we had to tell them he'll need a wheelchair.” (Mrs. C)
		“They have to focus on what's happening right now and they don't ask anything else.” (Mr. F)
		“…Sometimes they don't ask who is going to be with your son and does your son respond to that person which I think is important to know.” (Mrs. A)
	Having to Tell Them	“My experience is that we had to spend a lot of time trying to teach them [hospital staff] on how to deal with someone with developmental disabilities.” (Mr. E)
		“So when they would ask her questions and all she kept saying is going home, going home, it's obvious that that's all you're gonna get. I've had to explain to them this is how my sister communicates… They don't know how to… it seemed like the staff aren't facile and flexible and they don't know how to communicate as soon as they're thrown a monkey wrench. (Mrs. F)
	Support	“Alice lived in a residence they had [residence] staff stay with her… Truthfully, I had it kind of easy. Really if she hadn't been in a residence and I had to place her in the hospital I would've had to stay there, I never had to stay… I realized I was one of the lucky ones.” (Mrs. B)
		It was a little difficult because my son could not talk, okay but the [hospital] person was very nice and understanding, you know. I cannot really complain about them; they were proficient.” (Mr. II)
Need for Better Communication	Talking to the patient	“… With the nurses what we tried to do was to get them to understand who Bob was…We shouldn't talk of him as a third person that they should see him as a human being… So, we tried to instill in them to see Bob as another patient that was in need of medical care and that he was just like everybody else.” (Mr. E)
		“It's frustrating because people enter into the room and as soon as they see my sister they're very leery and they don't know how to approach her and many times what they do is they just don't talk to her…Interestingly enough, the physician in this particular hospitalization was phenomenal. I felt very comfortable with her, and I think the reason why I felt comfortable was my observation of how she treated my sister. She treated her like a human being, she spoke with her.” (Mrs. F)
		“Since he cannot communicate they have to, what you call it, they have to … No, they can't communicate with him, they communicate with me or the person who stays with him, like an aide.” (Mrs. H)
	Get the message across	“Post‐operatively he had a lot of distention and a lot of gas and the way he handled [expressed] that was to cry and cry and cry. So, he was in the PACU [Post Anesthesia Care Unit] and they were getting ready to close for the night and the nurse there said, ‘take your brother and his bellyache and go home’.” (Mrs. E)
		“Alice was just, truthfully, a very easy kid and happy and she would smile and that's all they needed to see… it wasn't charades but somehow she got her message across, believe me.” (Mrs. B)
	Talk to me	“…They seemed to be treating her as a delirious person, a behavioral issue. So her flailing around, her hitting, her whatever screaming she might have done, no matter how much we told them this is not her, she's never behaved this way. The emergency room doctor, you know, who's doing the examination and they weren't exactly comforting to either her or to me… I don't know how I would've reacted, but I probably would've trusted what the brother was saying. It was painful to watch; I was crying afterwards. I don't usually cry.” (Mr. J)
		“Hear what the person wants … to listen and to listen and to listen. To not prejudge, to take the time that's necessary to know that the family member may be a pain in the neck, I'm sure I was, but it's because the person, him or herself…is going through what they're going through, they're scared, they're in pain, they can't breathe, you know, whatever is wrong so the family member has the experience of feeling pain of their loved one and at the same time having to fight for them.” (Mrs. E)
	Work with me	“So I try to be, you know, as clear as I can to the nursing staff or the doctors with this is what my son takes, this is what he has to get. So usually, I would say for the most part my interaction with nurses has been positive and I think it's because I try to let them know, I'm here to help you help my son.” (Mrs. A)
		“…In some cases, I felt the staff was compassionate and understanding and really were accepting my communication on his behalf because my brother had very limited verbal abilities.” (Mrs. E)
		“I was scared because my sister didn't know what was going on… However, the surgeon, another physician was willing to work with me and let me go into the OR with her until she was out.” (Mrs. F)
Sense of Abandonment	Waiting	“We have spent overnight another day in the Emergency Room …Because I didn't want to spend another night, 2 nights in the Emergency Room and …is always very busy anyway.” (Mrs. A)
		“We were waiting six hours to admit him, in that six hours he decided he had to go to the bathroom…she handed him a urinal and stated call me when you finished … he is not able to handle a lot of things.” (Mrs. C)
	Just let them lay there	“…Every doctor that has treated Gene in the past 20 years says what a good patient he is. Sometimes that's not a good thing because you can just let them lay there and wait… because he's so good it's easy to just, we'll get to you, we'll get to you.” (Mrs. C)
		“… So, you know, it's like, don't make them second class citizens because they can't speak for themselves.” (Mr. J)
	Just leave me	“During the night…I try to stay awake but sometimes, you know I fall asleep and my son has actually taken out the IV there's blood all over and I assume that whoever is in charge, I'm there…it's okay but again, I'm a human being and if I fall asleep…I feel like it's like okay well the mother is there so let's just let her do it.” (Mrs. A)
		“I'm very easy, I'm very easy. I can understand everybody, what the problem is, you know. They're busy, there is somebody there. And for the most part the [group home] staff usually stays, you know … the nurses on the floor think that the staff person sitting there is maybe a private duty nurse … and the [group home] staff get very upset about that.” (Mrs. C)
	Need for extra care	“Well again there are rules hospitals have to follow, I understand, but there also should be some consideration of the special population that they're dealing with and that could be as simple as, you know, reaching out to the caregiver.” (Mr. J)
		“I would feel sorry for the patient because he doesn't understand that you have to stay there without having a glass of water…and you're there 6 hr, we had to sneak it to him, you know. So, I think that, you know, especially when they're disabled that they need that extra…Get them done and move them.” (Mrs. C)
		“Truthfully the last time she went to the hospital was when she did die and she really had regressed so much by then but everybody was so nice. She was in ICU almost the whole time she was there, that time she got extra care.” (Mrs. B)
Lack of Confidence	It happened	“Yes, it did happen. He takes a lot of medication…So I try to be as clear as I can to the nursing staff or the doctors with this is what my son takes…Vimpat 250 in the morning and he takes 300 of Vimpat in the evening and I think they reversed it. I feel if I were to just sit down and read a book or sleep while my son is in the bed mistakes could happen.” (Mrs. A)
		“So it's the lack of sensitivity, the way people say things, the lack of experience and then in other times, when he was immobile staff moved him like a sack of potatoes; he was short; he was heavy at times and they just threw him around.” (Mrs. E)
	I was scared	“What I wanted to do was establish a situation where the nurse would work with me first and try to show my sister what was going to happen, with IV's, etc., and when the nurse came in and I tried to say to her I'm my sister's legal guardian can I speak to you outside for a minute… She didn't wait for me she went down to the nurse's station and I heard her say to the other nurses ‘this one thinks she's some kind of doctor’ (Mrs. F)
		“Though both of those experiences were so frightening to me that never, ever, ever would I have her be alone, I was with her constantly in the hospital.” (Mrs. F)
		“Sometimes I feel the doctor doesn't care much for him because I don't know, because he doesn't speak or because…I don't know sometimes for some reason I don't feel comfortable…When he is in the hospital since he cannot speak he has to be tied up…They restrain him, that's the word, that, you know, affects me…It affects me when I see them restraining my son …and he can communicate with me.” (Mrs. H)
	Had not a clue	“In other cases people acting like they had not a clue how to work with somebody who was 60 + years and developmentally disabled…My brother also had Down's Syndrome and many health professionals still think that they should've died in infancy or childhood or young adulthood and are surprised to see them living as long as they are.” (Mrs. E)
		“It affects me when I see them restraining my son and I would like to…he can communicate with me, it's hard for them to examine him because they have to give him some injections… calm him down.” (Mrs. H)
	Put yourself in the place of the family	“If they feel themselves overworked, pressed if you will, you know, the easiest pushback is our population… so if I can't do my job you're making it too difficult…just picture if they had a child, a grandchild, sibling how they would want their sibling treated or at least cared for.” (Mr. J)
		“These situations are particularly complex and staff need to step back and really consider that, to kind of put themselves in the place of that family, that person and think about what it must be like, what it's doing to them.” (Mrs. E)

### 4.1 Need for advocacy

Advocacy required caregivers' presence due to patients' limited verbal ability. Crying could be a response to discomfort; meanwhile, silence may not indicate that all is well. Four subthemes emerged: “Have to be there”; “They don't ask”; “Having to tell them”; and “Support.”

#### 4.1.1 Have to be there

Hospitalization placed physical and emotional burdens on caregivers. Caregivers felt they had to be vigilant, resulting in the caregiver or a surrogate being constantly at the bedside.

#### 4.1.2 They don't ask

Several caregivers stated that nurses did not ask questions about patients' level of functioning and/or communication patterns.

#### 4.1.3 Having to tell them

Informing hospital staff about I/DD as a permanent condition was a common thread in the study. Specifically, caregivers revealed staff was poorly informed about older adults with I/DD. Statements implied the deficiencies were numerous.

#### 4.1.4 Support

Some caregivers noted residence personnel were better resources for information about their loved ones than hospital staff. However, there were situations where hospital staff provided adequate support.

### 4.2 Need for better communication

Patients with impaired verbal skills require communication tailored to their abilities. Four subthemes emerged from caregivers' descriptions: “Talking to the patient”; “Get the message across”; “Talk to me”; and “Work with me.”

#### 4.2.1 Talking to the patient

Several caregivers remarked staff did not address the patients because these individuals were presumed to be more limited than they were. Notably, one caregiver reported a very positive experience because a physician spoke directly to her sister.

#### 4.2.2 Get the message across

Some caregivers expressed staff was unable or unwilling to interpret patients' cues. However, one caregiver remarked hospital staff did correctly interpret her daughter's communication patterns.

#### 4.2.3 Talk to me

Several caregivers conveyed not receiving information about patients' care. One caregiver emphasized frustration at not being heard, leading to distress for himself and his sister. Another caregiver stressed the importance of listening to the caregiver.

#### 4.2.4 Work with me

Caregivers expressed healthcare professionals should include them in information sharing and decision‐making. Caregivers felt they had a wealth of information to share.

### 4.3 Sense of abandonment

Inattention to patient and caregiver needs left several caregivers with a sense of abandonment. Delays in the Emergency Departments compounded patient anxiety and behavioural problems. Four subthemes arose: “Waiting,” “Just let them lay there,” “Just leave me” and “Need for extra care.”

#### 4.3.1 Waiting

Waiting for extended periods led caregivers to believe staff was not tailoring care to patients. A caregiver transferred her son to another hospital to circumvent the wait.

#### 4.3.2 Just let them lay there

Caregivers interpreted delays as neglect. Many remarked that their loved ones were overlooked.

#### 4.3.3 Just leave me

Several caregivers reported feeling compelled to stay at the bedside even though they were exhausted. Caregivers also interpreted being left alone to provide care for the patient as being taken advantage of.

#### 4.3.4 Need for extra care

The need for extra care was especially important during admission. Of note, a caregiver reported extra care was afforded to her daughter because she was in the intensive care unit.

### 4.4 Lack of confidence

Several caregivers revealed a lack of trust in the hospital staff's ability to care for the patients. The subthemes were as follows: “It happened”; “I was scared”; “Had not a clue”; and “Put yourself in the place of the family.”

#### 4.4.1 It happened

Caregivers voiced complaints about medications, assessments and nursing care.

#### 4.4.2 I was scared

Various caregivers verbalized that communication issues compromised patient safety, causing caregivers to feel afraid and protective of their loved ones.

#### 4.4.3 Had not a clue

Some caregivers viewed the lack of experience of hospital staff as a lack of caring or knowledge.

#### 4.4.4 Put yourself in the place of the family

To understand their concerns, a few caregivers suggested hospital personnel demonstrate empathy. In summary, several caregivers reported encounters with hospital staff leaving them feeling isolated or afraid to the extent they had to stay with their loved ones. Other caregivers described the care received as not being equitable resulting in the perception that some hospital staff were biased and uncaring. Overall, caregivers expressed a lack of confidence in the staff's ability to care for adults with I/DD because of their communication experiences.

## 5 DISCUSSION

Family caregivers are considered experts on their family members, having in‐depth knowledge of patient needs; therefore, they have the ability to advise and support hospital personnel (Tuffrey‐Wijne et al., 2013). Facilitating caregivers in their roles as surrogate communicators improved patient safety and satisfaction (Larkin, Henwood, & Milne, 2018; Miller et al., 2016). For patients with I/DD, who generally have communication challenges, communicating with their caregivers is crucial (Larkin et al., 2018).

The current study was conducted to identify communication experiences of caregivers with hospital personnel; however, the data indicated issues primarily under the domain of nursing. For example, caregivers reported instances where a medication was not administered at the right time and pain assessments inadequately performed. Challenges confronted by caregivers included role confusion, patient safety concerns and a lack of trust in the hospital personnel's abilities.

Caregivers experienced frustration and role confusion when nurses expected them to perform nursing duties. Caregivers reported providing direct patient care, such as toileting, feeding and watching their loved ones to prevent them from falling or pulling out medical devices. Caregivers were untrained to perform nursing tasks, and their interventions could have led to negative outcomes. While some caregivers were expected to provide care, others were completely disregarded. Additionally, providing patient care caused physical and emotional effects on the caregivers. Some caregivers recounted having to take excessive time off from work or falling asleep at the bedside due to exhaustion.

Similar to the findings of Brolan et al. (2012), several caregivers in the present study believed they or a surrogate had to be present with the patients at all times to protect and advocate for them. Some of the caregivers reported medication not being administered at the prescribed time or improper handling of their loved ones. Subsequently, caregivers developed a generalized lack of trust in nurses' abilities to care for these patients unsupervised. Not only did caregivers face safety concerns, they also experienced negative attitudes and comments from some nurses. Such occurrences led caregivers to perceive nurses as being uncaring.

Findings of the current study were related to those of Lewis and Stenfert‐Kroese (2010). In that study, nurses admitted being more likely to rely on family caregivers to remain with the patient or help the staff. Caregivers in this study reported being left alone by hospital staff to provide care for their loved ones. In accord with most caregivers, Pelleboer‐Gunnink, Van Oorsouw, Van Weeghel, and Embregts (2017) revealed negative nursing staff attitudes as a cause of poor care. Caregivers were especially disturbed by their loved ones being ignored.

Negative staff attitudes have been reported as an underlying cause of poor or non‐existent communication with persons with I/DD (Iacono, Bigby, Unsworth, Douglas, & Fitzpatrick, 2014; Smeltzer et al., 2012). The current study found caregivers believed nurses did not speak to their family members because they did not see them as human beings worthy of care. Hence, caregivers developed a lack of trust in the nursing care their family members received. Rørtveit et al. (2015) advised that facilitating trust was related to communication, nurses being competent, interested, concerned and confident.

Expertise in the care of patients is a determinant in positive patient care outcomes (Boltz, Chippendale, Resnick, & Galvin, 2015). Healthcare professionals admitted to having deficiencies in knowledge, training and skills needed to care for individuals with I/DD (Hemm et al., 2015; Lewis, Gaffney, & Wilson, 2017; Redley et al., 2019; Smeltzer et al., 2012). At times, family caregivers in the current study were aware of staff's knowledge deficits. The lack of staff's proficiency with these patients left caregivers with a sense distrust.

Evidence‐based distrust in the staff's willingness and competence to care for this population was an undercurrent of this inquiry. Caregivers of this study implied that caring, compassion and empathy were important. Those components are some of the building blocks of trust. According to Richardson, Percy, and Hughes (2015), definitions of caring, compassion and empathy tend to be vague; nevertheless, family caregivers of this study recognized nurses who did not exhibit those traits. Several caregivers conveyed staff lacked empathy and desired that staff put themselves in the place of the family. The emotional toll of the hospitalization experience on the caregivers of this study cannot be overstated. Caregivers' feelings of anger, frustration, helplessness and grief were some of the factors that led them to try to protect their loved ones. Meanwhile, when trust was present in the nurse–caregiver relationship, caregivers reported feeling safer, more confident, heard and less angry.

Findings of this study and those of Miller et al. (2016) confirmed that when hospital staff communicated with family caregivers, it: (a) reduced caregivers' fears, (b) facilitated their ability to advocate on behalf of their family members and (c) improved their ability to provide comfort to their loved ones. Caregivers spoke about not having been asked the right questions about their loved ones, not having been heard, nor listened to. Caregivers' statements reflected their need for hospital staff to provide a supportive environment that would lessen the burden of care. This study highlighted including the family in the treatment plan, supporting them as caregivers, enhancing their access to information and acknowledging their contributions.

To summarize, the current study adds to the body of knowledge by providing family caregivers an opportunity to describe their experiences about communicating with hospital personnel. In addition, the study addressed an underrepresented area of nursing, namely, the care of hospitalized adults with intellectual and developmental disabilities.

## 6 LIMITATIONS

Most of the participants received services from the same agency, resided in adjacent counties and may have used the same healthcare facilities. Experiences may not have been conveyed accurately because of self‐report and loss of recall. Additionally, a dearth of prior research on this population made comparing data to previous studies difficult.

## 7 CONCLUSION

The current study was conducted to explore communication experiences of family caregivers of adults with intellectual and developmental disabilities and healthcare personnel during acute care hospitalization. Caregivers identified the importance of and barriers to communication between healthcare personnel, nurses, patients and themselves. Communication breakdowns heightened family caregivers' concerns about safety problems, potential for medication errors, poor pain management and toileting issues. These problems are the domains of nursing; consequently, caregivers felt they could not trust nurses to safely care for their loved ones. The need for nurses and other healthcare personnel to be educated on the care of this population and their families was evident throughout this study.

Findings also indicated the need to examine communication with this population and their caregivers from the nurse's perspective. Recommendations for practice include considering the patient and family caregivers as a unit, acknowledging the role and expertise of caregivers and incorporating them in the plan of care. Improving the care of this population involves educating nursing students and practicing nurses about adults with I/DD and their care needs. Moreover, emphasis should be placed on communication, listening, confidence building skills and advocacy.

## CONFLICT OF INTEREST

The author declares no conflicts of interest.

## Supporting information

Supplementary Material
