# Supplementary material for: Communication experiences of family caregivers of hospitalized adults with intellectual and developmental disabilities—A qualitative study
Source: Nurs Open. 2020 Jul 6;7(6):1725–34. doi: 10.1002/nop2.557 (PMC7544848; doi:10.1002/nop2.557)
Supplement: Supplementary file 1 — Supplementary Material [file NOP2-7-1725-s001.docx]

Appendix

Intellectual and Developmental Disabilities Caregiver Demographic Questionnaire

Your responses will be confidential and not associated with your name. Please create your own identification code by selecting 4 digits.

**Personal Identifier:**

*Subject selected code:*

**Demographics:**

*Age of Caregiver:*

*(a) 30-39*

*(b) 40-49*

*(c) 50-59*

*(d) 60-69*

*(e) 70 and above*

*Gender of caregiver*

*Age of I/DD Patient:*

*(a) 30-39*

*(b) 40-49*

*(c) 50-59*

*(d) 60-69*

*(e) 70 and above*

*Gender of I/DD Patient:*

*Ethnicity of caregiver:*

*Ethnicity of Patient:*

*Relationship to I/DD patient:*

*How long have you been the primary caregiver?*

*Does the patient live with you?*

*If the patient does not live with you, how often do you see him/her?*

*Caregiver signature: Date:*
